# Current Advances in 3D Tissue and Organ Reconstruction

**DOI:** 10.3390/ijms22020830

**Published:** 2021-01-15

**Authors:** Georgia Pennarossa, Sharon Arcuri, Teresina De Iorio, Fulvio Gandolfi, Tiziana A. L. Brevini

**Affiliations:** 1 Laboratory of Biomedical Embryology, Department of Health, Animal Science and Food Safety and Center for Stem Cell Research, Università degli Studi di Milano, Via Celoria 10, 20133 Milan, Italy; georgia.pennarossa@unimi.it (G.P.); Sharon.arcuri@unimi.it (S.A.); teresina.deiorio@unimi.it (T.D.I.); 2 Department of Agricultural and Environmental Sciences—Production, Landscape, Agroenergy and Center for Stem Cell Research, Università degli Studi di Milano, Via Celoria 2, 20133 Milan, Italy; fulvio.gandolfi@unimi.it

**Keywords:** tissue engineering, 3D matrices, biomechanical cues, microenvironment remodeling, hydrogel, micro-bioreactor, 3D printing and bioprinting, nanofiber-based scaffolds, decellularization, nanomedicine

## Abstract

Bi-dimensional culture systems have represented the most used method to study cell biology outside the body for over a century. Although they convey useful information, such systems may lose tissue-specific architecture, biomechanical effectors, and biochemical cues deriving from the native extracellular matrix, with significant alterations in several cellular functions and processes. Notably, the introduction of three-dimensional (3D) platforms that are able to re-create in vitro the structures of the native tissue, have overcome some of these issues, since they better mimic the in vivo milieu and reduce the gap between the cell culture ambient and the tissue environment. 3D culture systems are currently used in a broad range of studies, from cancer and stem cell biology, to drug testing and discovery. Here, we describe the mechanisms used by cells to perceive and respond to biomechanical cues and the main signaling pathways involved. We provide an overall perspective of the most recent 3D technologies. Given the breadth of the subject, we concentrate on the use of hydrogels, bioreactors, 3D printing and bioprinting, nanofiber-based scaffolds, and preparation of a decellularized bio-matrix. In addition, we report the possibility to combine the use of 3D cultures with functionalized nanoparticles to obtain highly predictive in vitro models for use in the nanomedicine field.

## 1. How to Overcome the Hurdles of 2D Cell Culture Systems

In vitro two-dimensional (2D) culture systems have represented, till recently, the most widely used strategy to study the mechanisms underlying cell biology, as well as diseases and drug action outside the body [1]. The first 2D approach was developed in 1907 by Harrison and colleagues to maintain nerve fibers in culture [2]. Subsequently, this method was applied for the study of different cell types, ranging from pluripotent and/or multipotent stem cells to terminally differentiated adult cells. The major advantages of 2D systems are associated with the simple and low-cost maintenance of cell cultures, the fast-downstream processing, and the easy performance of functional tests [1]. During the years, several steps forwards have been made to ameliorate and turn this technique into a more flexible and quicker platform. An example is represented by the modification and functionalization of the plastic surface onto which cells are seeded, using different materials and/or proteins, in order to resemble certain microenvironments [3]. Nevertheless, these improvements were not sufficient to mimic the natural structures of the original tissue and cells continued to be cultured in a monolayer bidirectional manner, providing sub-optimal conditions for their growth and specific functions. This negatively influences the fundamental cellular features and, usually, compromises the viability and reliability of the experiments, as well as the correct understanding of the whole organ activity [1,4]. Indeed, cells isolated from tissues and transferred onto flat and hard plastic substrates have been shown to lose their specific phenotype, because of the absence of a tissue-specific architecture and of the naturally occurring biomechanical and biochemical cues that cannot be mimicked in 2D in vitro systems. In particular, one of the most compromising aspects is represented by the alterations and/or the complete loss of the cell-to-cell and cell-to-extracellular matrix interactions. This leads to significant changes in several cellular features and processes, including cell morphology, polarity, differentiation, proliferation, genetic pattern, responsiveness to stimuli and secretions, drug metabolism, and many other functions [5,6,7,8,9].

The recent biotechnological advances have partly overcome these hurdles, thanks to the use of three-dimensional (3D) culture systems, which more closely mimic the natural in vivo milieu. The 3D culture methods are currently used in a broad range of in vitro studies, including cancer and stem cell biology, and drug testing and discovery. The first pioneering attempt to produce a 3D culture model dates back to the 1970s, when Hamburg and Salmon used a solution of soft agar to embed and grow human single cells [10]. Since then, several cutting-edge techniques were developed, ranging from hydrogels to organoid models and synthetic or biological scaffolds. The common key aspect of these new approaches is represented by their ability to in vitro recreate the macro- and micro-architecture of tissues and organs, encouraging cells to re-organize in complex 3D structures that closely resemble the in vivo micro-topography. The resulting in vitro environment replicates the biochemical and biomechanical effectors, directly influencing both cell fate and behavior.

In this review, we describe the fundamental mechanosensing-related cues that enable cells to integrate and cooperate with the extracellular microenvironment. We provide an overview of the recent progress in cell culturing that implements the use of 3D models, including hydrogels, bioreactors, 3D printing and bioprinting, and preparation of decellularized bio-scaffolds.

## 2. Biomechanical Sensors and Effectors of the Microenvironment

The cell microenvironment is regulated by several factors, which include not only temperature, pH, oxygen, metabolites, growth factors or peptides, and hormones, but also macro- and micro-architecture-related cues as well as stretching and contracting stimuli. Externally applied forces are directly controlled by the stiffness of the substrate that the cells adhere to [11] and result in biomechanical signals that have a broad impact on cell behavior [12,13]. Cells are indeed able to sense the surrounding microenvironment and to interact with it, regulating their shape, their intracellular organization, growth, and differentiation, as well as their functionality. Interestingly enough, this ability is not limited to somatic cells and has emerged as an active property of oocytes and early embryos, which may actively sense biomechanical stimuli and convert them into intracellular signals, tuning their own behavior. In particular, in the context of the female gamete, the two main mechanosensing signaling pathways, namely Hippo and RhoGTPase, have been demonstrated to be involved in many fundamental oogenesis processes and to influence oocyte quality. Indeed, while the RhoGTPase signaling pathways are important actors in the coordination of actin filaments and microtubule formation, as well as polar body extrusion and spindle rotation during meiosis, the main actors of the Hippo pathway, Yes-associated protein (YAP) and WW domain-containing transcription regulator protein 1 (WWTR1 or TAZ), have an essential role during oogenesis, are highly transcribed in mouse and human oocytes, are maternally accumulated, and have been reported as strong candidates for zygotic genome activation [14].

**Figure 1:**
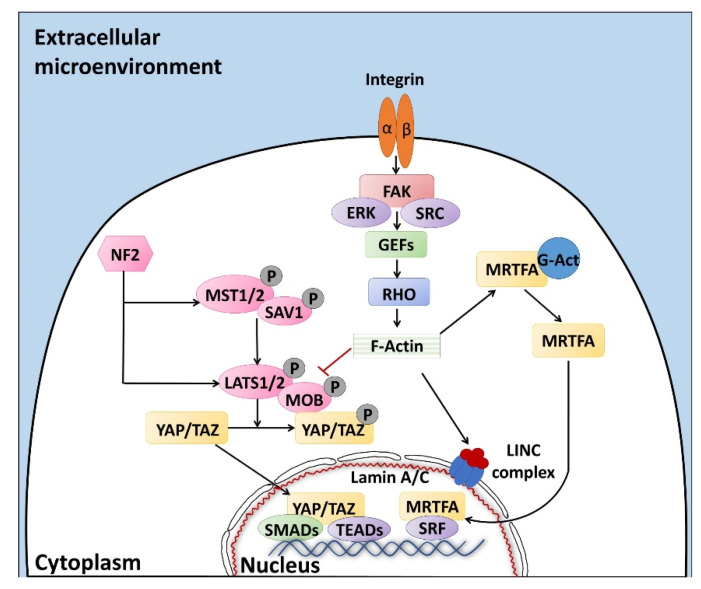
The sensors (integrins) and effectors (Rho and Hippo pathways) driving mechano-transduction processes, regulating YAP/TAZ localization and the subsequent cell behavior. Signaling pathways are described in detail in the text. Black arrow: activation, red T bar: inhibition of phosphorylation and molecule activation.

Altogether, the properties described above are defined with the term “mechano-transduction”, which indicates the cellular mechanisms by which mechanical inputs, such as stretching, tension or fluid flow, are converted into intracellular signals [15]. They are regulated by several membrane proteins that modify their folding in response to extracellular biomechanical stimuli [16]. Integrins have been recognized as the main molecular link between cells and the extracellular matrix (ECM). This protein family is characterized by the ability to sense, respond to, and bidirectionally interact with the cellular environment [17]. More in detail, integrin’s key function is to trigger the cytoplasmatic recruitment and activation of adaptors and signaling proteins, such as Protein Tyrosine Kinase 2 (PTK2B, also known as FAK), SRC Proto-Oncogene Non-Receptor Tyrosine Kinase (SRC), and Mitogen-Activated Protein Kinase 1 (MAPK1, known as ERK), which assemble into the focal adhesion complex [18]. Force-mediated formation and/or reorganization of focal adhesion complexes, in turn, modify the Rho family small GTPase activities, leading to enhanced actin polymerization and the formation of stress fibers. This latter event can produce two different effects, described as short- or long-range transmission of force. The first directly affects the nuclear localization and function of the mechanosensitive transcription regulators, such as Myocardin Related Transcription Factor A (MRTFA, also known as MKL1) and YAP/TAZ [19]. The long-range effect transmits the force from the surrounding environment into the cell nucleus, thanks to a physical actin-mediated connection between the ECM and the linker of the nucleoskeleton and cytoskeleton (LINC) complex (Figure 1) [20]. This results in several changes, including chromatin remodeling, exposure of specific sites to transcription factors, gene expression changes, and the modifications of nuclear pore conformation and size, finally promoting YAP/TAZ nuclear translocation.

YAP/TAZ represent the main downstream effectors of the Hippo pathway cascade [21], which is involved in the cellular response to extracellular stimuli. Their compartmentalization is strictly correlated to mechanical signals, cellular stress, polarity, and adhesion cues [20], and influences gene expression, thus controlling cell fate. Recent studies also demonstrate the presence of upstream molecules, such as mammalian STE20-like protein kinase 1/2 (MST1/2), Salvador family WW domain containing protein 1 (SAV1), MOB kinase activator 1A/B (MOB1A/B), and large tumor suppressor 1/2 (LATS1/2), which regulate the Hippo pathway activity [22,23,24]. In particular, it has been shown that phosphorylation events are involved in the activation of the cascade, inducing cytoplasmatic retention of YAP/TAZ (Figure 1). In contrast, when the cascade is inactive, these two proteins translocate into the nucleus, interacting with different transcription factors (TFs). It is interesting to note that the YAP/TAZ subcellular localization is tightly controlled by substrate rigidity and topography [25,26,27], as well as by actin remodeling [28,29,30] and cell stretching [28]. This clearly demonstrates a specific role of these molecules as mechanotransducers and mechanosensors. In agreement with this, cells grown on a 3D soft matrix have been demonstrated to display YAP/TAZ localized to the cytoplasm, whereas cells plated on standard plastic supports (2D system) showed YAP/TAZ localized to the nucleus [26]. In addition, substrate stiffness is able to modulate the expression of the two molecules, which are upregulated in cells grown on hard hydrogels compared to soft substrates [31].

Furthermore, we recently demonstrated that YAP/TAZ localization is mirrored by a parallel compartmentalization of SMAD Family Member 2/3 (SMAD2/3). In particular, we observed that cells with cytoplasmic retention of YAP/TAZ exhibited a SMAD2/3 cytoplasmic distribution, while cells displaying the two molecules localized in the nucleus showed concomitant SMAD2/3 nuclear accumulation [23]. This latter event led to the formation of the YAP/TAZ–SMAD2/3 complex, which is known to bind to TEA domain transcription factors (TEAD), regulating cell fate and differentiation processes [32], both in bi-parental and mono-parental parthenogenetic cell lines [33,34,35].

It is indeed important to note that YAP/TAZ are transcriptional coactivators, unable to directly interact with DNA, but rather binding other TFs in order to elicit their functions [36]. Accumulating evidences point, in particular, to the TEAD protein family as the main actor, mediating YAP and TAZ nuclear activity and modulating the expression of different target genes involved in cell growth, proliferation, and organ development [23,37].

## 3. D Cell Culture Systems

The cellular microenvironment is a highly complex 3D natural structure, regulating in vivo cell functional activities through specific stimuli [38,39,40,41]. As a consequence, the organization and composition of the ECM, as well as the contribution of cell-to-cell and cell-to-ECM interactions, play a fundamental role in the control of cell behaviors and are strictly tied to the functions of tissues and whole organs. 

The major aim of the recently introduced 3D cell culture systems is to model and in vitro re-create the tissue and organ in vivo milieu, mimicking the underlying biochemical and biomechanical signals [42,43]. A well-selected and -designed microenvironment can be therefore used in tissue and cell engineering to faithfully re-produce in vitro the conditions that promote all cell functions, including proliferation, migration, matrix production, and stem cell differentiation. The main 3D cell culture strategies that are available to this end are shortly described in the sections below. 

### 3.1. Hydrogels

The physiological relevance of Matrigel remained undisputed for many years. This material was considered the gold-standard support for 3D cell culture systems and, therefore, used in multiple applications, ranging from angiogenesis assays to organoid assembly and pluripotent embryonic stem cells expansion. However, due to its natural origin, Matrigel is inherently limited by its compositional complexity and lack of batch-to-batch reproducibility. Furthermore, the introduction of exogenous components by Matrigel interferes with mechanistic studies of cell behavior and limits its therapeutic applications [44]. Powerful alternatives were thus provided by the introduction of the chemically defined synthetic hydrogels that show minimal batch-to-batch variation, increased reproducibility, defined material properties, compositions, and controllable degradation properties [45,46]. Notably, Matrigel has recently found innovative applications, such as the reconstruction of 3D and hollow-shaped functional units of glandular tissues in a controlled microenvironment. This is achieved thanks to the development of a set of scaffolds (hemispheres, Matrigel beads, open and closed microtubes, or microcarriers) that serve as building blocks for a 3D toolbox aimed at producing such 3D constructs [47].

The term “hydrogel” was used for the first time in 1894 to describe a colloidal gel consisting of inorganic salt [48]. Hydrogels received considerable attention in the past 50 years and several materials displaying high swelling ability and good mechanical properties were developed for a various range of applications, including hygienic products, agriculture, drug delivery systems, sealing, coal dewatering, artificial snow, food additives, pharmaceuticals, biomedical applications, tissue engineering and regenerative medicines, diagnostics, wound dressing, separation of biomolecules or cells and barrier materials to regulate biological adhesions, and biosensors [49].

Hydrogels or hydrophilic gels are biomaterials consisting of a water-swollen network of physically or chemically crosslinked polymers [50]. They can be composed of chains of natural polymers (collagen, fibrin, or alginate), synthetic polymers (polyvinyl alcohol, polyacrylic acid, or polyethylene glycol), or hybrid materials, which combine elements of synthetic and natural polymers (hyaluronic acid and polypeptides). Depending on the polymeric composition, as well as on the nature and density of the network joints, hydrogels can contain various amounts of water and, consequently, can display different degrees of flexibility. Indeed, the cross-linking processes used for their synthesis also influence the mechanical properties of the supports. In particular, hydrogels may be synthesized in a number of ways, including chemical cross-linking, thermal annealing, supramolecular self-assembly, ionic gelation, and electrostatic interactions [51]. Based on the variation in polymer concentrations and cross-linking methods, it is possible to tune the physical characteristics of the scaffolds, namely, their architecture, stiffness, pore size, diffusion of soluble factors, and ligand density [52].

The pivotal study that first demonstrated the use of hydrogels for producing cell culture supports is, however, more recent—dating back to 2006. In this manuscript, Engler et al. demonstrated that the substrate stiffness and elasticity directly influence cell fate as well as lineage specification [53]. Currently, a great number of hydrogels with different “stiffness” is commercially available. The term “stiffness” is used to indicate how a component bends under load, while still returning to its original shape once the load is removed. It is associated with the elastic deformation and directly depends on the modulus of elasticity, also known as Young’s modulus of the material. In the field of biology and tissue engineering, ECM composition and elasticity regulate the Young’s modulus of the extracellular microenvironment, which, in turn, plays a fundamental role in regulating cell behavior. These specific in vivo features are completely lost in 2D culture systems that use standard plastic supports, characterized by elevated stiffnesses. In contrast, hydrogels allow to better mimic native tissues and cellular environments, modulating the elasticity and stiffness. This aspect together with the hydrogel’s enormous versatility, as well as their biocompatibility and viscoelastic properties, make them highly suitable for 3D culture support production. At present, the hydrogels are used in a wide variety of biological applications [54], including tissue engineering, micro-organ systems, drug delivery, cytotoxicity testing, and drug screening. Several tissue and cell types have been in vitro cultured using this technology, including cartilage [55,56], cornea [57,58], skin [59], tendon [60], vascular tissue [61], pancreatic endocrine cells [62], and pluripotent cells [63].

One potential drawback in hydrogel use for tissue engineering-related applications could be the employment of harmful crosslinkers and the lack of appropriate degradability. Several studies are currently in progress in order to carefully address these critical points and develop polymerization methods that allow for the production of safe and biocompatible hydrogels.

### 3.2. Micro-Bioreactors

Keller et al. described the first in vitro culture method encouraging the formation of spheroid structures [64]. In this system, known as “hanging drop”, cells are suspended in a droplet of medium and are allowed to self-aggregate under the influence of gravitational forces. It is interesting to note that reproducibility, production efficiency, and organoid size uniformity are common aspects in all 3D spheroid-based applications [65]. Nevertheless, the hanging-drop technique consists of a stationary system, primarily based on diffusion-limited conditions, which usually results in the formation of loose-aggregated clusters. Indeed, cell suspension deposition onto the underside of the lid of a tissue culture dish leads to the creation of a microgravity environment that concentrates cells at the free liquid–air interface, inducing the generation of low-aggregated multicellular spheres. Based on this, during the last decades, several studies were focused on the development of novel and more efficient in vitro culture models that maximize cell-to-cell interactions, promote self-assembly, and encourage the formation of organoids.

**Figure 2:**
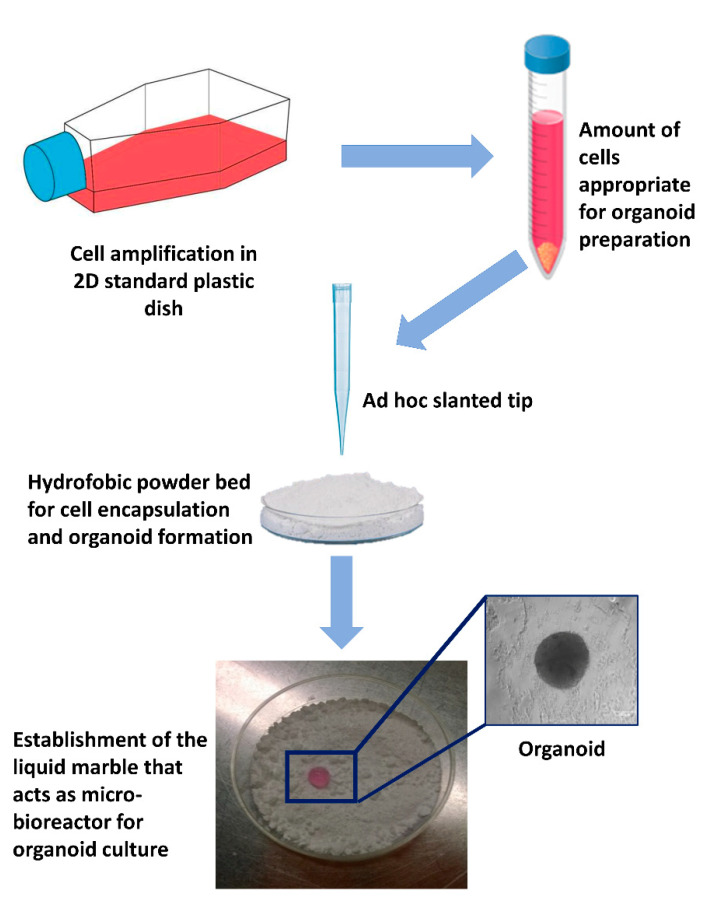
Schematic representation of the protocol used to produce micro-bioreactors and obtain spheroid organoids. 2D cultured cells are detached for the dish and centrifuged. Droplets of medium containing cells are dispensed onto the hydrophobic powder bed. The powder covers the drop, leading to the creation of the liquid marble that allows cell aggregation and organoid formation.

To date, very promising results were generated encapsulating cells in Matrigel microbeads, acting as a single-cell culture compartment and serving as microcarriers for flow-based high-throughput analysis, opening novel possibilities for performing high throughput screens on 3D culture structures in biological and pharmaceutical research [66]. In parallel, other studies demonstrated the successful use of 3D scaffold-free models, consisting of a small liquid droplet coated with a hydrophobic powder that eliminates the direct contact between the interior liquid and the external environment. The idea of encapsulating cells resuspended in a liquid with hydrophobic powder was derived from gall-dwelling aphids [67], where the honeydew waste secreted from the aphid is covered with a powdery wax before being rolled away, preventing it from drowning the insects. The first microbioreactor, also known as “liquid marble”, was reported by Aussillous et al., who covered a water droplet with hydrophobic microparticles [68]. Subsequently, several other studies demonstrated the significant advantages offered by the use of microreactors, compared to other culture approaches. First of all, these systems provide a non-adherent liquid surface that induces rapid cell aggregation and the generation of compact spheroids (Figure 2). Furthermore, the concave bottom, the spherical shape, and the internal liquid flow, distinctive of the liquid marbles, allow cells to settle onto the bottom of the micro-bioreactor, forming organoids uniform in size and shape [65]. Liquid marbles also display a remarkably high surface/volume ratio, which enhances the mass and heat transfer, increasing the reaction kinetics and improving the yield, and an optimal gas exchange that can occur through the entire surface. Lastly, the use of micro-bioreactors allows for a reduction in reagents and volumes, a more precise control over reaction parameters, and a safer handling of hazardous or exothermic reactions [69,70,71,72]. Thanks to all these unique properties, together with the easy manipulation and the high reproducibility and efficiency, liquid marbles have found numerous applications in the field of microfluidics, storage, transportation, and mechanical computation [73,74,75,76], as well as in the chemical and biological areas [77]. Recently, several studies have pointed to micro-bioreactors as ideal platforms, essential for biomedical applications and culture of microorganisms or cells of different types, spanning from oocyte [78], cardiac [79], pancreatic [80], toroid [81], and olfactory ensheathing [82] to high plasticity cells [32]. In particular, we demonstrated that cell encapsulation in micro-bioreactors induces distinctive 3D cell rearrangements and specific cell-to-cell interactions [32]. In parallel, in our experience, the use of micro-bioreactors significantly enhance the acquisition and maintenance of a high plasticity state, providing a useful platform in stem cell organoid technology for long-term culture of different cell types, ranging from epigenetically erased cells to embryonic stem cells (ESCs) and induced pluripotent stem cells (iPSCs), as well as mesenchymal stem cells (MSCs) [65]. Interestingly, parallel studies also demonstrated that encapsulation systems can be successfully used to store and cryopreserve organoids without altering cell growth and differentiation ability, as well as to perform post-treatments, such as direct transfection, and post-genetic screen and organoid analysis [83].

### 3.3. 3D Printing Scaffolds

3D printing is an additive manufacturing technique for fabricating a wide range of objects with complex geometries. The first 3D printer was created in 1986 by Charles W. Hull, who indicated with the term “stereolithography” the method and the machinery used for producing solid structures by a gradual accumulation of thin layers of material, one on top of the other [84]. Subsequently, the term “3D printing” was coined and used to describe a work done in 1993, in which a standard inkjet printer was modified to a custom processing apparatus [85].

This technique was immediately considered of particular interest due to its great potential in the production of reliable and reproducible scaffolds, suitable for tissue engineering application.

**Figure 3:**
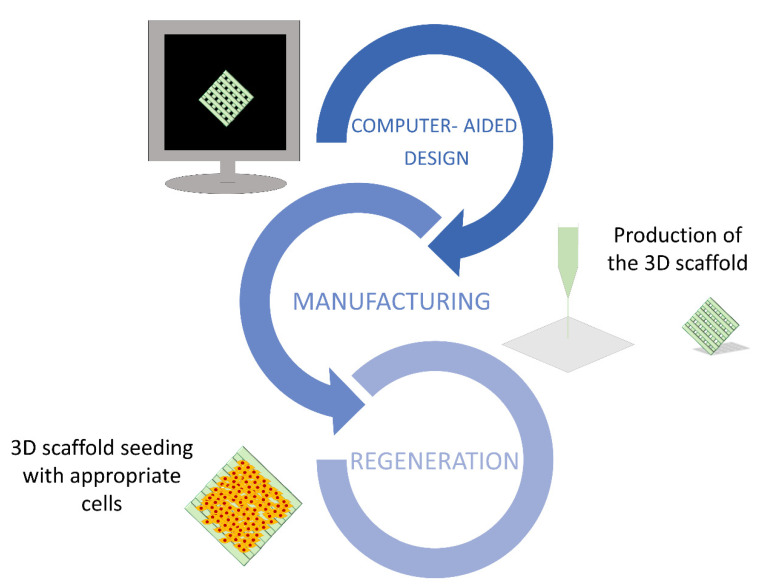
The main fundamental steps needed to construct an artificial organ in vitro. The 3D scaffold shape is modeled using computer-aided design (CAD) software (design). The support is 3D printed after an accurate selection of the most adequate method (manufacturing). Lastly, it is repopulated with the specific cell type/types (regeneration).

The fabrication of 3D supports is based on a consecutive deposition of material layer by layer, or even pixel by pixel. The shape of the scaffold is firstly modeled using computer-aided design (CAD) software, such as UG, CATIA, Tinkercad, or ProE, in order to produce a file containing all the information required to control the moving track of the printing device (Figure 3). The most adequate 3D printing method need then to be selected among the different technologies currently available. These are categorized into three different groups, namely, powder-, ink-, and polymerization-based. The most common approach involves the use of a continuous filament of thermoplastic polymers that are heated at the nozzle to reach a semi-liquid state and, then, extruded on the platform or on top of previously printed layers (fused deposition modelling).

Although the post-manufacturing processes, such as heat treatments and hot isostatic pressing, can tailor the general characteristics of the scaffolds, including microstructure and surface roughness [86], the material selection remains a fundamental step. Indeed, the physical properties, cytotoxicity, bioactivity, and biodegradability significantly influence the mechanical features and the performance of the scaffolds. To date, several materials and biomaterials have been developed and proposed for tissue engineering applications. Among them, the most common used are the biodegradable natural and synthetic polymers, such as poly lactic acid, polycaprolactone, polyglycolic acid, or their copolymers [87,88,89]. In contrast, although non-degradable ceramics, such as alumina and zirconia, represent excellent candidates for joint replacement, they cannot be successfully adopted in other tissue engineering areas, because of their biological inertness [90]. This problem can be solved by using hydroxyapatite and other bioactive glasses composed by silicon dioxide and calcium oxide, which have shown to possess both high mechanical and bioactivity properties [91]. In contrast, metals are the least used in tissue engineering scaffolding due to their low biocompatibility, with the exception of titanium and its alloys, which have high bioactivity and biocompatibility [92].

During the last years, particular emphasis was also given to 3D bioprinting, which applies a layer-by-layer concept, but, unlike standard 3D printing, deposits cell-laden filaments or cell-containing droplets into specific substrates, in order to generate 3D biological structures [93,94]. Currently, there are different bioprinting strategies are available, namely, inkjet-based bioprinting, laser-assisted (LAB) bioprinting, pressure-assisted (extrusion) based bioprinting, acoustic bioprinting, stereolithography (SLA)-based bioprinting, and magnetic bioprinting [95,96]. These techniques can be used alone or in combination, depending on the tissue type to be reconstructed. Many factors affect printing fidelity and influence the overall porosity, mechanical strength, and layer height of the scaffold, including applied pressure, printing speed, and printing distance [97]. Another working parameter to be critically considered is represented by the solution of the biomaterial loaded with the desired cell types. This is usually referred to as “bioink” and its appropriate selection represents a crucial aspect for developing functional tissue or organ constructs. To date, several natural and synthetic polymers have been used in bioprinting techniques, including alginate, collagen, silk, dextran, gelatin, fibrin, agarose-chitosan, agarose, gellan gum, hyaluronic acid, decellularized matrix, matrigel, hydroxyapatite, polyethylene glycol, methacrylated hyaluronic acid, polyvinylpyrrolidone, poly-epta-caprolactone, pluronic, polyglycidol-hyaluronic acid, and polyhydroxybutyrate. The selection of the appropriate polymer is carried out based on the specific cell/tissue to be used.

A fascinating application of the 3D printing technology was also used for the creation of innovative models for medical purposes. In particular, anatomically accurate internal organ images, obtained with X-rays, computed tomography, dual-energy imaging, cone beam computed tomography, or digital tomosynthesis, can be printed in order to produce modular anthropomorphic mannequins [98]. This allow for replicating and studying a wide range of pathologies [99,100,101,102,103], as well as for reducing the ethical concerns [100,102,103] relative to the use of cadaveric and animal models for education, surgical planning, and training purposes.

Currently, the development and optimization of novel materials, combined with reduced manufacturing costs and advanced printers, enabled the use of 3D printing in many research laboratories as well as in industrial settings [104,105,106]. Nevertheless, although 3D printing techniques allowed to in vitro re-create several tissues, such as bone, cartilage, skin, nerve, and complex organs, like teeth, nose, ears, heart, and liver, there are few studies utilizing 3D-printed scaffolds in preclinical models and trials. This is mainly related to technical drawbacks that limit the transfer from bench to bedside of 3D printing techniques, possibly caused by poor cell survival and differentiation, insufficient vascularization rate, and altered metabolite diffusion. In order to solve these issues, several translational studies are currently in progress to overcome the problems mentioned above and eventually ensure a smooth transfer of knowledge acquired at the laboratory benches to the patient bed sites.

### 3.4. Nanofiber-Based Electrospun Scaffolds

The recent development of nanofibers has attracted attention and led to significant improvement in the production of scaffolds that can replicate the architecture of natural tissues at the nanoscale [107]. It is well known that natural ECM consists of several interwoven protein fibers, with variable diameters ranging from 10 to 100 nm [108], which modulate cell adhesion as well as their growth and behavior [109].

To date, different techniques have been developed to fabricate nanofibrous scaffolds, including phase separation [110], self-assembly [110], electrospinning [111], liquid-assisted collection [112], and template-assisted collection [113]. Among all these approaches, electrospinning is considered one the most promising process, thanks to its ability to generate fibers, which are similar to those of native ECM, obtained with the use a wide range of materials [107]. A great advantage is also represented by the observation that electrospun nanofibers display a large surface area, as well as their porous structure that significantly favor cell adhesion, proliferation, migration, and differentiation [107]. A variety of natural polymers, such as collagen, gelatin, elastin, and silk, as well as synthetic materials, such as polylactic acid, polyglycolic acid, polycaprolactone, and polylactic-co-glycolic acid, have been used as biomimetic substrates. However, it must be noted that the use of synthetic or natural polymers alone have shown important limitations [114]. Indeed, synthetic polymers have great flexibility in synthesis and modification, while they lack cell affinity due to the low hydrophilicity and the absence of surface cell recognition sites [109]. On the other hand, natural polymers provide good biocompatibility, but display poor processing ability and mechanical properties [115]. It is therefore desirable to produce scaffolds using composite nanofibers that possess not only suitable structural properties, but also bioactive surfaces [116]. In addition, when required, nanofibers can be further functionalized via incorporation of bioactive molecules, including enzymes, DNA, and growth factors [117].

Nanofibers can be electrospun in various patterns, depending on the biomedical application required. It is therefore fundamental to know the structural and biological properties of tissue in order to choose the materials and the fiber orientation that best fit the 3D reconstruction. Indeed, electrospun nanofibrous scaffolds can have different alignments, such as axial, yarn, and radial, providing an increased ability in shaping cell morphology, guiding cell migration, and encouraging cell differentiation, compared to other types of scaffolds [118,119]. Currently, several strategies have been developed for controlling nanofiber alignment. Overall, these methods can be classified into three major categories, namely, mechanical, electrostatic, and magnetic, depending on the type of forces involved [120,121,122,123].

This approach has been used for in vitro reconstruction of different structures, including vascular [124,125,126,127], neural [128,129,130,131], bone [132,133,134,135,136], cartilage [137,138,139,140], and tendon/ligament [141,142,143,144,145] tissues. Nevertheless, despite the recent advances in creating biomimetic nanofibrous scaffolds for tissue engineering applications, several issues still remain unsolved. One of the most challenging aspect is related to the complexity of creating 3D porous scaffolds, combined with cells and growth factors, to optimize the engrafting and viability of the cells. It is also crucial to develop novel strategies for producing fibers with a diameter similar to that of native tissue (usually less than 100 nm, preferably in the range of 10–50 nm). These fibers must display high porosity, which is necessary for cell infiltration and migration [117]. Several studies are currently in progress to address these problems. Among them, the electro-spinning/netting technique appeared to be very promising and seems a versatile method for the generation of spider-web-like nano-nets with an ultrafine fiber diameter (less than 50 nm), maintaining a high porosity [109]. Last but not least, it is important to highlight that the majority of the published studies report results generated in vitro. It is now mandatory to confirm the potential of this technique in vivo, further optimizing nanofiber scaffold composition and structure and, as a consequence, translating electrospun nanofiber production from the laboratory dimension to the industrial scale.

### 3.5. Decellularized Scaffolds

A promising alternative is represented by the creation of natural bio-scaffolds through a recently developed method that involves the complete decellularization of an organ. The process is based on the removal of the cellular components from the tissues to obtain an acellular ECM, also known as a “decellularized scaffold”. This approach involves the use of different strategies that allow the removal of native cells and genetic materials, while preserving ECM with its structural, biochemical, and biomechanical properties. Decellularization can be performed through chemical, enzymatic, physical, or combinative techniques. The chemical approach can involve the use of surfactants, acids (i.e., peracetic acid), or bases (i.e., sodium hydroxide), which are able to solubilize the cell membrane and nuclear material. Among the several surfactants adopted for disarranging the phospholipid membranes, sodium dodecyl sulfate (SDS) represents the most effective decellularizing agent, successfully used for several tissue and organs, including forearm [146], cornea [147], myocardium [148], heart valve [149], small intestine [150], kidney [151], vein [152], lungs [153,154], ovary [155], and heart [156]. However, it has been demonstrated that this compound can induce damages to the structural and signaling proteins, such as collagen [149], glycosaminoglycans (GAGs), and growth factors [157], preventing the full retention of ECM biomechanical and biochemical properties. It is therefore imperative to choose and develop specific protocols that limit the concentration and the exposure time for this agent. In parallel, different studies also indicated that Triton X-100 and sodium deoxycholate (SD) could represent promising substitutes for SDS, thanks to their low aggressiveness that allow for an efficient cell removal, while preserving the ECM [150,153,157,158].

Enzymatic approaches, including DNase I, RNase, or trypsin/ethylenediaminetetraacetic acid (EDTA), can be used to remove the nucleic acid residuals after cell rupture or to target the peptides that anchor cells to the ECM. These enzymes are usually inefficient when applied alone and need to be used in combination, in order to supplement the chemicals’ activities. Furthermore, a protracted exposure to enzymes may result in the alteration of collagen, laminin, fibronectin, elastin, and GAGs, damaging the ECM microstructure.

Lastly, the physical and mechanical methods, such as freeze–thaw cycles (freezing temperatures around −80 °C and biological temperatures around 37 °C), high hydrostatic pressure (higher than 600 MPa), supercritical carbon dioxide (CO_2_, higher than the critical temperature of 31.1 °C and the critical pressure of 7.40 MPa), and nonthermal permanent electroporation, can also be used in decellularization protocols. These approaches remove tissue constituent cells and genetic materials, while maintaining the ECM architecture and composition, combining cell lysis and cell-matrix adhesive protein disruption.

It is important to highlight that all the approaches listed above show advantages and limitations. For example, the mechanical methods are usually less damaging to the tissue structure, but they fail to eliminate genetic materials. On the other hand, surfactants at low concentrations or enzymes used alone may not completely remove all cellular debris. As a consequence, several protocols present in the literature combine two or more types of treatment, in order to complement one another, with the final goal of retaining the ECM’s desired characteristics and yield an optimal decellularized scaffold. In our experience, the use of a four-step protocol, involving a freeze–thaw cycle, followed by sequential incubations with SDS, Triton X-100, and deoxycholate, allow to obtain whole-ovary bio-scaffolds that successfully preserve the shape, architecture, and ECM of the original organ [156]. We also demonstrate that the obtained ECM-based scaffold recreates in vitro the complex in vivo ovarian milieu, facilitating the necessary interactions between cells and their surroundings and ensuring a correct cell growth, differentiation, and function [158].

While there are no official guidelines for an adequate decellularization, standard metrics are beginning to emerge. It has been recently proposed that at least four parameters need to be met in order to assess the achievement of a proper decellularization process:

1. cell removal: no visible nuclear material by histological H&E and 4′,6-diamidino-2-phenylindole (DAPI) staining;
2. elimination of genetic material: less than 50 ng double-stranded DNA (dsDNA) per mg ECM dry weight and less than 200 bp DNA fragment length;
3. preservation of structural protein (i.e., collagen, fibronectin, and laminin), GAGs and growth factor content;
4. retention of mechanical properties, including elastic modulus and tensile strength.

The tissue decellularization technique can be applied to any organ. To date, different protocols have been developed for a variety of tissues, as reported in Table 1. However, no conclusive evidence is available and further improvements are mandatory to optimize methods for obtaining acellular ECMs that fully preserve the structural, biochemical, and biomechanical properties of the native tissue. Furthermore, the resulting scaffolds need to be reproducible, sterile, and successfully undergo long-term storage for future procedures. These advances, together with the development of scaled-up methods, are crucial to produce bio-scaffolds that can be of use in tissue engineering and, more in general, in regenerative medicine applications.

## 4. Tissue and Organ Regeneration Approaches

**Figure 4:**
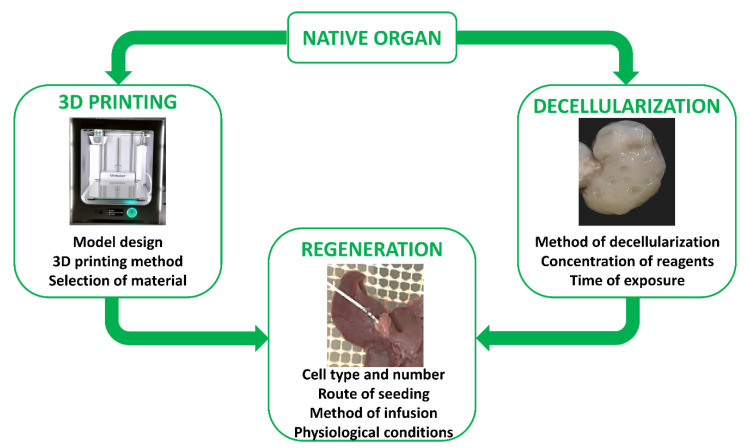
The two main ways and essential key aspects to successfully recreate in vitro a fully functional bio-artificial organ.

The first reports describing in vitro skin reconstruction date back to over 30 years ago [173,174,175,176], while the first bio-artificial bone tissue was developed in the 1990s by Ishaug et al. [177] and Baksh et al. [178]. Following these pioneering studies, extraordinary progress has been made in tissue engineering, not only for the optimization of scaffold production processes, but also for the regeneration and recellularization methods [179,180]. Indeed, cell repopulation plays a key role in getting fully functional bio-artificial organs. Needless to say, this procedure needs to be improved and optimized, and above all, adapted to the specific biophysical and biomechanical proprieties of the scaffold used as well as to the complexity of the tissue or organ of interest. Indeed, each organ varies in its unique structural components, namely, its cell types, matrix, architecture, as well as in its biophysical and biochemical environment, such as pressure, flow, oxygen tension, cytokines, and growth factors. As a consequence, the repopulation step requires the use of appropriate specific cell types, while commanding an optimized seeding method and a physiologically relevant culture approach (Figure 4). Skin cell sheet or blood vessel in vitro re-creation, for example, requires a single cell type, whereas whole organ reconstruction necessitates the seeding of multiple cell types to reestablish all the functional structures, including parenchyma, vasculature, and supporting components.

To date, distinct cell sources, such as embryonic stem cells, mesenchymal stem cells, fetal and adult tissue cells, and induced pluripotent cells, have been used to repopulate both artificial scaffolds as well as biological ones. The first tissue-engineered airway was transplanted into a patient in 2008. In this work, the authors repopulated a trachea scaffold with epithelial cells and mesenchymal stem-cell-derived chondrocytes. The results obtained demonstrated that it was possible to in vitro re-create a tissue-engineered trachea with mechanical properties that allow normal functioning, providing successful treatment for patients [166]. Following this, many studies using a single type of tissue-specific stem cells [158,170,181,182,183,184,185] or induced pluripotent cells [185,186,187,188] were carried out, and others are currently ongoing, in order to identify the best 3D supports able to boost cell differentiation and successfully produce in vitro functional tissues and organs for future possible transplantation. In parallel, both synthetic and biological scaffolds were repopulated with terminally differentiated cells, including fibroblasts [155], mature hepatocytes [189,190,191], and pancreatic endocrine [192,193,194], cardiac [195], and ovarian cells [196].

Nevertheless, it is still difficult to identify and standardize common protocols indicating routes of delivery, methods of infusion, and the correct cell number to be applied for the generation of functional organs. The main limitations are currently related to the complexity and the specialization of tissues, and, in some instances, to the elevated experimental costs. Indeed, although, different studies have already described distinct approaches obtaining encouraging results, none of them have proved their absolute potential to replace a damaged organ or tissue and several issues need to be solved.

In the kidney, a variety of successful strategies were developed [197]. However, the current techniques still show important limitations that impede the obtainment of a complete and functional whole-organ. In particular, while a piecemeal of renal components were reconstructed in vitro, the nephron structure has not yet been recreated [198].

In contrast, the liver represents a simpler, more feasible model, thanks to its regular structure and clear cellular organization. The current technical approaches in liver tissue engineering are based on the use of different methods, such as cell encapsulation, 3D printing, 3D bioprinting, and decellularized organs, which display the appropriate topography and biomechanical properties that facilitate hepatocyte colonization, migration, differentiation, proliferation, and cell polarity [199,200,201]. It is, however, important to highlight that, to date, 3D liver tissue organization and function has been mostly demonstrated in animal models and further studies and evidence are necessary in order to confirm the generation of a functional liver for human transplantation.

Many strategies were also developed for 3D heart recreation. Nevertheless, they led to the production of functionally immature contractile cardiac constructs [202,203,204]. Furthermore, although cardiomyocytes represent the key cell type of this tissue, the engrafting of stromal and endothelial populations, for the recreation of vascular system, remains the most immediate need for obtaining functional organs [198].

Lastly, the lung represents an extraordinary challenge to engineer almost from scratch. The studies carried out up to now demonstrated the recreation of some microscopic aspects of alveolar organization, without any gas exchange [205,206,207]. This organ, in fact, displays a unique and complicated architecture and is composed of more than 40 different highly specialized cell types. Furthermore, the peculiar structure of each capillary vessel, surrounded on both sides by a one cell-thick epithelium, which is needed to ensure carbon dioxide–oxygen exchange, represents the greatest obstacle for successful lung tissue engineering [205].

## 5. Combining 3D Cell Culture Systems with Nanoparticles for the Creation of Predictive Models

During the last two decades, research in the nanotechnology field has undergone a fast development. Following the first work by Brus [208], inorganic nanoparticles (NPs) have been largely investigated for diverse applications in biology and medicine, ranging from NP-based therapies [209] to biosensors [210]. NPs can display different shapes, such as spherical [211,212], rod-shaped [213,214], wire [215], plane [216,217], star [218], cage [219], and multipod [220,221], and are generally described as particulate material characterized by dimensions ranging from 1 to 100 nm [222,223]. They possess several peculiar properties, namely, a high surface-to-volume ratio, elevated chemical reactivity and dispersibility, significant surface energy, and unique mechanical, thermal, electrical, magnetic, and optical behaviors [224]. All these features make NPs highly promising for nanomedicine applications. Indeed, the delivery of small and macro molecules using nanoparticulate vehicles offers a number of advantages compared to the administration alone [225], solving the main issues related to poor bioavailability, impaired target specificity, and systemic and organ toxicity [226,227,228].

The earlier examples of nanomedicine involved the use of lipid- and polymer-based nanocarriers for drug encapsulation and delivery [229,230,231]. Subsequently, innovative techniques involving the use of NPs were developed for biosensing [232], stem-cell modulation [233], and tissue engineering [234].

**Figure 5:**
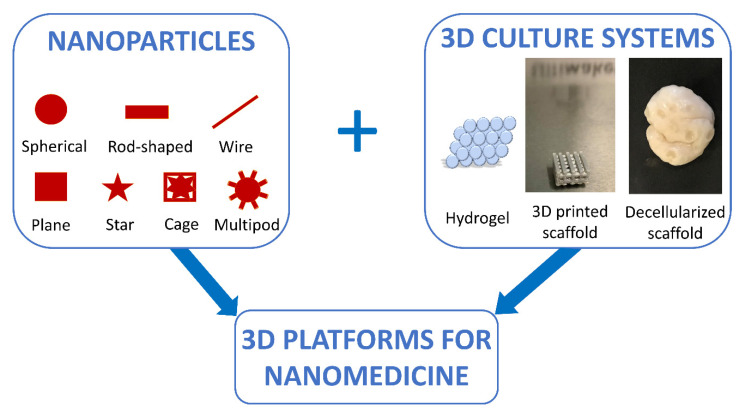
Nanomedicine approaches are improved through a combined use of NP-based techniques and 3D culture systems.

The evaluation of NP delivery is currently being carried out in 3D culture systems because of the 2D culture approach’s failure to account for the extracellular microenvironment that is present in vivo [235]. This also allows for studying how the interactions with ECM components can affect fate and stability of the NPs. In addition, this approach makes it possible to understand how the mechanical forces, such as tension, compression, and interstitial fluid flow, can alter the bioavailability of the delivery vehicle, by redirecting the NPs in the direction of flow and potentially away from the target sites. Furthermore, it is important to remember that cell culture conditions also affect the cell phenotype and thereby influence the cellular response to the delivered drugs. In particular, multicellular drug resistance, possibly caused by the occurrence of hypoxic tissue regions, is able to alter cell proliferation, and poor availability of delivered drugs in deeper tissue layers [236] can be only mimicked in 3D culture conditions. For these reasons, researchers are combining the use of 3D culture systems with nanomedicine, with the final goal to design better predictive in vitro models (Figure 5). Reproducible in vitro 3D tissue culture systems that more closely mimic in vivo tissues, in terms of functionality, morphological, and mechanical architecture, could be applied during the development and evaluation of delivery vehicles, to offer a more predictive platform for assessing in vivo delivery efficiencies. Such systems can serve to bridge the considerable gap between traditional 2D monolayer experiments, animal studies, and clinical trials, since NP formulations can be more effectively optimized in 3D in vitro models.

NPs were, for example, combined with the use of micro-bioreactors to investigate their accumulation and penetration in spheroid organoids and in solid tumors [237,238]. Using this 3D model, different studies demonstrated that smaller particles can penetrate deeper into 3D multicellular structures [239]. This led researchers to develop novel size-switching NPs, contributing to the advance of nanotheranostics [240]. Indeed, NPs are being investigated for use in photothermal therapy as theranostic agents. Currently, ongoing preclinical in vitro studies involve the use of 3D culture systems to analyze the efficacy of NP-based therapy [235].

It is also interesting to note that, although scaffold-free 3D models are suitable for studying the penetration of NPs in multicellular organoids, this method lacks the complexity of an organ with its parenchyma and stromal-supporting components, which the NPs have to pass through in an in vivo setting. To overcome this problem and verify the influence of the ECM on NP internalization, it is possible to use the so called “scaffold-embedded models”, namely hydrogel (as described above), to display an architecture and mechanical properties that closely resemble a natural ECM.

Presently, one of the most interesting approaches is represented by the use of bioengineered 3D models to produce in vitro platforms that replicate host–pathogen early interactions and offer a powerful insight into the possible strategies to prevent virus entry into the cell. In this context, we recently described a model to investigate early infection events in the upper airway [241]. More in detail, we created a synthetic hydrogel-based 3D culture system that mimics in vitro the complex architectures and mechanical cues distinctive of the upper airway epithelia. We then exposed the in vitro-generated 3D nasal and tracheal epithelia to virus-mimicking gold NPs (AuNPs), displaying the typical shape and size distinctive of SARS-CoV-2 and of the majority of Coronaviridae presently known. This infection platform, beside its predictive power and flexibility, is aimed also at ensuring safety. In our understanding, this represents a crucial aspect, since dealing with a virus that is highly contagious, virulent, and even deadly, inevitably reduces the manpower and the number of laboratories that have high protection facilities, significantly affecting in a way the intellectual and scientific resources to fight this as well as other future pandemics [242].

Although a completely different prospect, an innovative nanomedicine approach can be found in the use of thrombospondins (TSPs) to remodel the cell microenvironment. TSP is an ECM protein family, with the ability to modulate multiple physiological and pathological events in healthy tissues as well as in tumors, including cell growth and spreading, angiogenesis, inflammation, and immunity. In addition, it has been recently demonstrated that TSPs play a fundamental role in tissue remodeling, influencing the extracellular microenvironment properties and composition and regulating the interactions between the ECM structural proteins and cells [243]. Based on this, the use of TSPs in nanomedicine can be useful to modify the tumor microenvironment and regulate the progression of cancer growth, which is strictly related to the vascular and ECM remodeling as well as the inflammatory response [244,245]. It must be noted, however, that the complex nature of TSP interactions and functions, including their different cell and tissue specific effects, have led to confusing results and controversial conclusions and it is still debated where these proteins might become desirable targets for an integrative anti-cancer approach.

## 6. Conclusions and Perspectives

The research of the last years have clearly demonstrated that traditional in vitro 2D culture systems do not faithfully imitate the physiological and biochemical features of cells in their original tissue. Indeed, growing evidence shows significant differences between the microenvironment and the effectors provided by bi-dimensional in vitro cell culture models and those of in vivo tissues. This may greatly alter cell response and behavior, in turn heavily affecting the translation of results from bench to bedside, and inevitably leading to the use of large numbers of experimental animal testing, in order to gain reproducible and reliable data.

All these aspects eventually have an impact on the applications of the results, greatly affecting the final outcome of the research. A good example is provided by the high attrition rate that the pharmaceutical industry has been struggling with over the past years when trying to introduce a new prescription medicine into the market [246], only gaining marketing approval after astronomic costs, largely due to unsatisfactory, misleading testing models during the early pre-clinical phase [247,248].

Altogether, this clearly points to the need for continuous improvement of our in vitro models, in an attempt to ensure their physiological meaning and predictive capability. The powerful experimental strategies discussed in the present manuscript all have the common aim to exploit combinations of mechanosensing-related effectors and chemical cues. This can be generated by modulating substrate stiffness, printing functional scaffolds, or preparing a decellularized biological matrix. Regardless of the approach selected, the result will allow for a fine-tuning of the in vitro micro- and macro-environment that produces organized arrangements of cells and encourages the derivation of a well-defined, physiologically organized tissue structure, improving/leading to the acquisition of a specific cell phenotype.

Selecting the most appropriate culture system that best mimic the in vivo situation would lead to the generation of refined models that more closely mimic the physiological environment, enhancing the predictive power of the culture system. This, in turn, would allow for a reduction in the number of animals used to translate the results in vivo, emphasizing the concept of non-invasive investigations and animal welfare and protection.

## Figures and Tables

**Table 1:** Overview of the protocols developed for the decellularization of organ fragments and whole organs.

**Organ Fragments**	**Protocol**	**Reference**
Porcine testis	−80 °C, 0.01% SDS, 1% Triton X-100	[159]
Goat lung	0.25%Trypsin-EDTA, 0.1% SDS, 20 μg/mL RNase A and 0.2 mg/mL DNase I	[160]
Human lung	8 mM CHAPS ^1^	[154]
Porcine myocardium	0.1% SDS, 0.01% Trypsin, 1 mM PMSF ^2^, 20 μg/mL RNase A and 0.2 mg/mL DNase I	[161]
Porcine liver	0.01% SDS, 1% Triton X-100	[162]
Porcine kidney	1% Triton X-100	[163]
**Whole Organ**	**Protocol**	**Reference**
Bovine ovaries	0,1% SDS	[164]
Porcine ovaries	−80 °C, 0.5% SDS, 1% Triton X-100, 2% SD	[155]
Ovine testis	1% SDS	[165]
Human trachea	4% SD, 2000 kU DNase I in 1 mM NaCl	[166]
Porcine trachea	4% SD, 2 kU/mL DNase I in 1 M NaCl	[167]
Porcine lung	−80 °C, 2% SDS, 1% SDS	[168]
Human lung	1% Triton X-100, 0.5% SDS	[153]
Porcine heart	0.2% Trypsin/0.05% EDTA/0.05% NaN_3_, 3% Triton X-100/0.05% EDTA/0.05% NaN_3_, 4% SD	[169]
Rabbit liver	ddH_2_O, 1% Triton X/0.1% NH_4_OH	[170]
Porcine liver	1% SDS, 1% Triton X-100, 1% SD	[171]
Porcine cornea	0.5% SDS	[147]
Goat kidney	50 U/mL heparin, 0.1% SDS, 0.5% SDS, 1% SDS, 0.1% Triton X-100, 5 mM CaCl_2_ and MgSO_4_, 0.2 mg/mL DNase I	[172]

^1^ CHAPS = 3-((3-Cholamidopropyl)-dimethylammonio)-1-propanesulfonate. ^2^ PMSF = phenylmethylsulfonylfluoride.

## Abbreviations

2D	Two-dimensional
3D	Three-dimensional
ECM	Extracellular matrix
FAK	Protein Tyrosine Kinase 2
SRC	SRC Proto-Oncogene Non-Receptor Tyrosine Kinase
ERK	Mitogen-Activated Protein Kinase 1
MRTFA	Myocardin Related Transcription Factor A
YAP	Yes-associated protein
TAZ	WW domain-containing transcription regulator protein 1
LINC	Linker of nucleoskeleton and cytoskeleton
MST1/2	STE20-like protein kinase ½
SAV1	Salvador family WW domain containing protein 1
MOB1A/B	MOB kinase activator 1A/B
LATS1/2	Large tumor suppressor 1/2
TFs	Transcription factors
SMAD2/3	SMAD Family Member 2/3
TEAD	TEA Domain Transcription Factor
ESCs	Embryonic stem cells
iPSCs	Induced Pluripotent Stem Cells
MSCs	Mesenchymal Stem Cells
CAD	Computer-aided design
SDS	Sodium dodecyl sulfate
GAGs	Glycosaminoglycans
SD	Sodium deoxycholate
EDTA	Ethylenediaminetetraacetic acid
H&E	Hematoxylin and Eosin
DAPI	4′,6-diamidino-2-phenylindole
CHAPS	3-((3-Cholamidopropyl)-dimethylammonio)-1-propanesulfonate
PMSF	Phenylmethylsulfonylfluoride
NPs	Nanoparticles
AuNPs	Gold nanoparticles
